# Rationale and design of the Anal HPV, HIV and Aging (AHHA) study: Protocol for a prospective study of anal HPV infection and HSIL among men who have sex (MSM) or trans women living with and without HIV, ages 50 and older

**DOI:** 10.3389/fepid.2022.992718

**Published:** 2022-11-16

**Authors:** Alexandra L. Hernandez, Christopher Scott Weatherly, Ryan Gonzalez, Sepideh Farhat, Maria Da Costa, Joanne Calderon, Jason Kauffman, Arezou Sadighi Akha, Joan F. Hilton, Joel M. Palefsky

**Affiliations:** ^1^Department of Medicine, University of California, San Francisco, San Francisco, CA, United States; ^2^Public Health Program, College of Education and Health Sciences, Touro University, Vallejo, CA, United States; ^3^College of Osteopathic Medicine, Touro University Nevada, Henderson, NV, United States; ^4^Department of Epidemiology and Biostatistics, University of California, San Francisco, San Francisco, CA, United States

**Keywords:** human papillomavirus, HPV, HIV, aging, anal cancer, HSIL, men who have sex with men, MSM

## Abstract

**Introduction:**

More than half of people living with HIV in the US are 50+ years old. Despite the benefits of antiretroviral therapy, older individuals with HIV are at higher risk for illnesses than their HIV-negative counterparts. Anal cancer, anal high-grade squamous intraepithelial lesions (HSIL), and anal HPV-16 infection occur most frequently among men who have sex with men living with HIV (MSMLWH). Men aged 60+ are 3-fold more likely to be diagnosed with anal cancer compared with younger men. Despite the increasing risk of anal cancer with age and HIV, little is known about the relationships among aging, HPV infection, HSIL and HIV.

**Methods and analysis:**

The Anal HPV, HIV, and Aging (AHHA) Study is a two-stage project to evaluate the relationships among anal HPV infection, HSIL, HIV infection, and biomarkers of biological aging in MSM or trans women over the age of 50 years. Stage 1 will estimate the cross-sectional prevalence of both anal HPV infection and HSIL, based on outcomes of anal HPV DNA testing, and high-resolution anoscopy with biopsy. We will also study associations with study outcomes and serological biomarkers of inflammation (interleukin-6, C-reactive protein, D-dimer) and with the Veterans Aging Cohort Study Index and the Fried Frailty Phenotype using multivariable models. Participants living with HIV (*n* = 150) and HIV-negative participants (*n* = 150) will be enrolled. The 3-year Stage 2 longitudinal sample restricted to HSIL-negative and anal HPV-16 DNA-negative participants will estimate the 3-year incidence of both anal HSIL and anal HPV, stratified by HIV status through Cox proportional hazards regression. The effect of biomarkers of inflammation and markers of aging on study outcomes will be evaluated through multivariable repeated measures models stratified by HIV status.

**Ethics and dissemination:**

This protocol was approved by the University of California, San Francisco Institutional Review Board (IRB: 16-18966). Results will be disseminated through presentations at national/international scientific conferences and publication in peer-reviewed journals.

## Introduction

Effective antiretroviral therapy (ART) has changed the lives of people living with HIV (PLWH) by transforming HIV into a chronic manageable condition. Growing numbers of PLWH are reaching older ages and it is estimated that over half of the PLWH in the U.S. are now over the age of 50 (1). While the advent of effective ART has lengthened life for PLWH it has not restored full health (2). Several illnesses associated with advanced age, including a number of cancers (2–4), are now common among PLWH receiving ART. These diseases may also be occurring at younger ages among PLWH than among their HIV-negative counterparts (5). In particular, the role of aging in the pathogenesis of anal cancer among MSM living with HIV (MSMLWH) is poorly understood.

PLWH are at particularly high risk of anal cancer. A population-based study in the U.S. reported that anal cancer was the fourth most common non-AIDS-defining cancer among PLWH (6). Ninety-seven percent of anal cancer cases were considered excess cases when compared to the general population (6). The incidence of anal cancer among MSMLWH [85–131/100,000] (5, 7) is substantially higher than among HIV-negative MSM [up to 37/100,000] (8) and as compared to men in the general population [1.6/100,000] (9). Development of anal cancer is preceded by anal high-grade squamous intraepithelial lesions (HSIL) (10) and both anal cancer and HSIL are understood to be caused by anal HPV infection, particularly HPV-16 (11), similarly to cervical cancer. A recent study has shown that treatment of anal HSIL prevents the occurrence of anal cancer (12) just as treatment of cervical precancerous lesions prevents cervical cancer (13).

Among HIV-negative men, anal cancer incidence increases with age and men aged 60 years or older are three times more likely to be diagnosed with anal cancer when compared with men of younger ages (14). There may be a synergistic effect of age and HIV infection, and older MSMLWH may have an even higher incidence of anal cancer than what would be expected given their age and HIV status alone, but this has not yet been evaluated.

Many of the abnormalities seen in PLWH and older individuals are likely to be at least partially related to chronic inflammation. Untreated PLWH have high levels of inflammation measured by increased inflammatory cytokines or coagulation factors and most of these levels are reduced with appropriate ART (4, 15, 16). However, a number of markers of inflammation remain elevated even among PLWH who are successfully treated (17, 18). Markers of inflammation that remain elevated include interleukin-6 (IL-6), C-reactive protein (CRP), and D-dimer (17), and have been associated with all-cause mortality (19) and opportunistic infections (18). IL-6, CRP, and D-dimer have also been shown to have associations with cancer in both PLWH and HIV-negative populations (20, 21).

The mechanism through which aging impacts cancer is not fully understood and other measures of aging apart from chronic inflammation may be useful as biomarkers for biological aging. The Veterans Aging Cohort Study (VACS) Index (22, 23) combines data from 2 research evaluations and 3 self-report domains, including blood tests, age, and body mass index (BMI) and has been associated with all-cause mortality among PLWH (24). The Fried Frailty Phenotype (FFP) (25) has been validated as a clinical syndrome in elderly HIV-negative populations and predicts loss of independence, disability, falls, and mortality (25–29).

The Anal HPV, HIV and Aging (AHHA) Study provides first steps in evaluating associations of two measures of aging and three serological biomarkers of inflammation with prevalent and incident anal HSIL and anal HPV infection. We hypothesize that the biomarkers of aging evaluated in this study will be increased with increasing chronological age, HIV infection, HPV infection, and HSIL. We expect that this study will elucidate potential pathways between HPV infection to anal cancer that could, in turn, be the focus of future interventions to prevent anal cancer, and potentially other cancers associated with aging and HIV.

## Methods and analysis

### Study design and objectives

The overall goal of the AHHA Study is to evaluate the associations of anal HPV infection, anal HSIL, HIV infection, and biological aging in MSM and trans women 50 years old and older. The study is a two-stage project.

The goal of Stage 1 is to estimate the prevalence of anal HPV and HSIL by HIV status among MSM and trans women ages 50 years and older. During Stage 1, a cross-sectional study, we are recruiting 300 individuals to estimate the prevalences, by HIV status, of anal HPV infection and anal HSIL. We also will evaluate associations of these outcomes with chronological age and biomarkers of inflammation and aging.

#### Objective 1.1

To estimate the type-specific prevalence of anal HPV infection by HIV status and its association with chronological age and biomarkers of inflammation and aging among MSM and trans women aged 50 years and older.

#### Objective 1.2

To estimate the prevalence of biopsy-confirmed anal HSIL by HIV status and its association with anal HPV infection, chronological age, and biomarkers of inflammation and aging among MSM and trans women aged 50 years and older.

**Figure 1:**
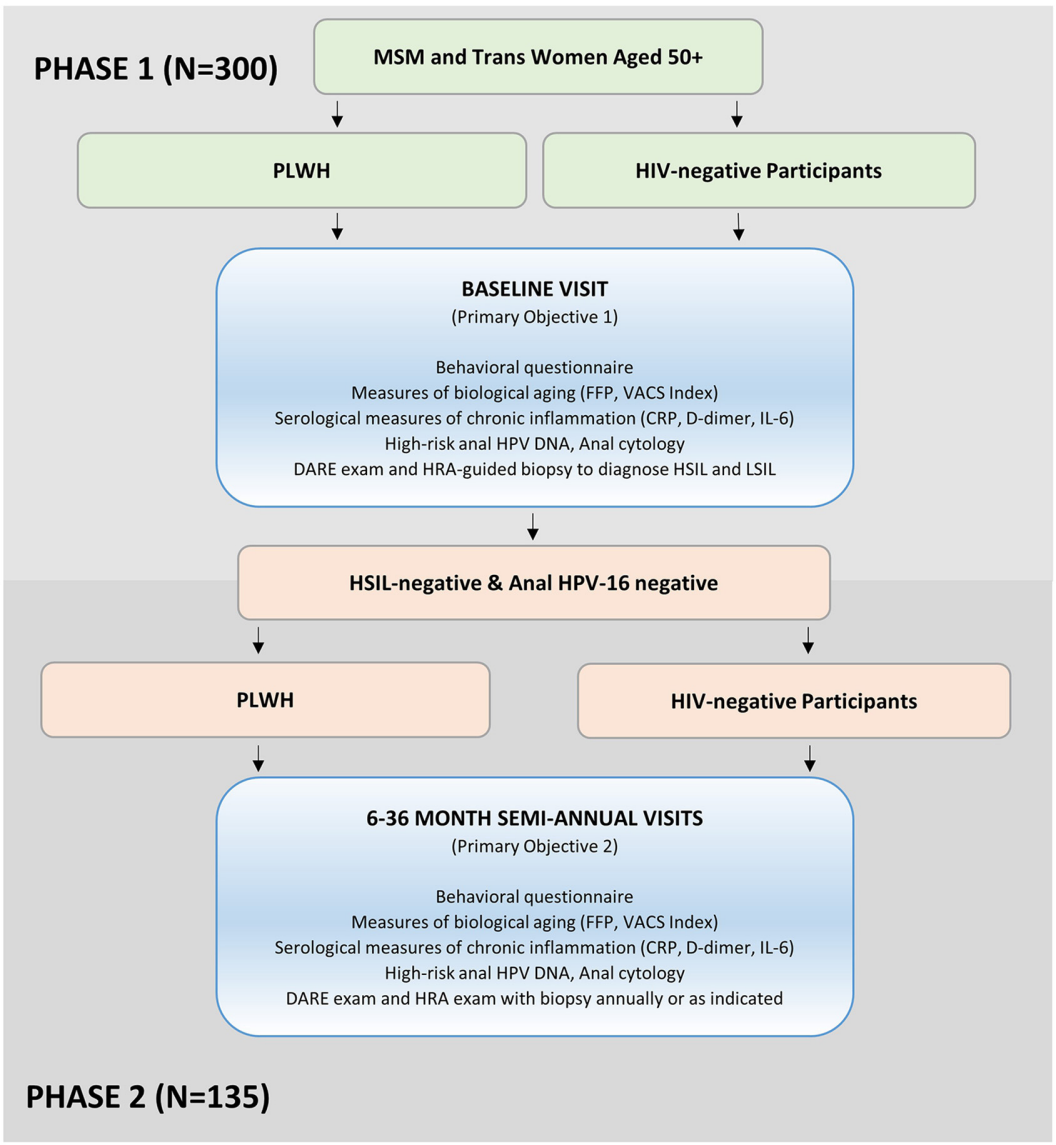
The AHHA study participant recruitment and enrollment flowchart. MSM, men who have sex with men; PLWH, participants living with HIV; FFP, fried Frailty Phenotype; VACS Index, Veterans Aging Cohort Study Index; CRP, c-reactive protein; IL-6, interleukin-6; DARE, digital anorectal examination; HRA, high-resolution anoscopy; HSIL, high-grade squamous intraepithelial lesions; LSIL, low-grade squamous intraepithelial lesions.

The goal of Stage 2 is to estimate the incidence of anal HPV and HSIL by HIV status among MSM and trans women ages 50 years and older. During Stage 2, we are prospectively following two cohorts of Stage 1 participants who were anal HPV-16 DNA-negative and did not have a diagnosis of anal HSIL. All participants in Stage 2 participated in Stage 1. Every 6 months for 3 years we are evaluating 45 individuals who are living with HIV and 90 individuals who are HIV-negative (Figure 1). Complete follow-up includes 7 study visits over the 3-year period.

#### Objective 2.1

To estimate the incidence of anal HPV-16 infection in anal HPV-16-negative and HSIL-negative participants, by HIV status, and its relationship to chronological age and biomarkers of inflammation and aging among MSM and trans women ages 50 years and older.

#### Objective 2.2

To estimate the incidence of anal HSIL in anal HPV-16-negative and HSIL-negative participants by HIV status and its relationship to chronological age and biomarkers of inflammation and aging among MSM and trans women ages 50 years and older.

#### Objective 2.3

To describe the type-specific HPV DNA present in incident anal HSIL biopsy samples among MSM and trans women ages 50 years and older.

### Setting and timeline

The AHHA Study is taking place at the University of California, San Francisco (UCSF) within the Anal Neoplasia Clinic Research and Education (ANCRE) Center. The ANCRE Center was founded in 1990, by Principal Investigator Joel Palefsky, and is the world's first clinic devoted to prevention of anal cancer (30). Enrollment for the AHHA Study began in September 2017. The study rational and protocol was presented to the UCSF Center for AIDS Prevention Studies Community Advisory Board (CAPS-CAB) in July of 2018 to request suggestions and feedback.

## Study population

### Inclusion and exclusion criteria

Individuals are eligible for the AHHA Study if they identify as a man or trans-person who has sex with men (MSM) and are aged 50 years or older. Individuals must be willing and able to attend study visits at the ANCRE Center every 6 months for 3 years to be eligible for inclusion. Individuals who cannot provide informed consent are not eligible for the study. Individuals with a history of anal cancer, screening for anal cancer, and/or treatment or removal of HSIL are ineligible for AHHA. In addition, those who are HSIL-positive and anal HPV-16 DNA-positive at baseline are not eligible for AHHA's Stage 2 prospective cohort study.

### Recruitment and enrollment

In Stage 1, we are currently enrolling two convenience cohorts of 150 HIV-negative participants and 150 participants living with HIV (PLWH) into the AHHA Study (Table 1).

**Table 1:** Recruitment strategies for the AHHA Study for MSM in the San Francisco Bay Area.

**Activity**	**Description**
Recruitment focus groups	Five focus groups with men in our target population were conducted to determine how to motivate older MSM to participate in research, where to advertise to older MSM, and how to reach MSM through social groups or social media.
Print advertisement	Custom flyers, palm cards, posters, and banners are being posted in targeted community organizations, doctor's offices, and in various additional community settings.
Referral from health care provider	Our dedicated outreach worker is working to build a network of health care providers in the community who serve PLWH as well as individuals over the age of 50. We will collaborate with these providers enlisting their assistance to refer men or trans women who have sex with men to our study.
Community outreach	Dedicated outreach building relationships with members of the community providing information on HPV infection, HPV vaccination, HPV-associated pre-cancer, and HPV-associated cancer as well as soliciting direct referrals from community organizations.
Social media, email lists, and dating apps	AHHA Facebook and website with direct email link for enrollment. Advertisement on electronic email lists, and other community websites, dating applications (ex. Scruff, Grindr) that target men in our target population, Facebook, and Craigslist.
Modified snowball sampling	All participants are given the opportunity to refer up to five friends and/or acquaintances to the study.

MSM, Men who have sex with men; App, application or program for phones, tablets, or other mobile devices.

Several different methods are being used to identify, recruit, and enroll our study population by a dedicated recruitment and retention specialist. We are recruiting through referrals, physical advertisements placed throughout the community, and *via* an online advertisement campaign. We also ask each participant to refer up to five candidate participants–friends or acquaintances–using a modified snowball sampling method (31, 32).

We expect 90 (60%) of HIV-negative and 45 (30%) of PLWH will be HSIL-negative and anal HPV-16 DNA-negative at baseline, thus eligible for Stage 2 longitudinal follow-up.

Participants are reimbursed for their time (visits range from 1.5 to 3 h in length) and travel with $100 per visit. Stage 1 only participants can be reimbursed up to $100 and Stage 2 participants can be reimbursed up to $700.

### Participant retention

The target population is generally highly motivated and eager to contribute to their community. We are using similar procedures previously used at the ANCRE Center to ensure that participants return for their follow-up visits. This includes creating a safe, caring environment for participants and collecting detailed contact information from each participant. We have a comprehensive protocol for participant retention, including text message reminders 1 week and 1 day before study visits and ongoing contact *via* website blog entries and Facebook posts. We also call all participants with missed visits to reschedule them.

## Stage 1—Cross-sectional study to address objectives 1.1 and 1.2

All 300 participants who enroll in the AHHA Study will complete a baseline study visit (Figure 1, Table 2). At their first visit, the potential participant completes informed consent procedures and confirms eligibility. If the participant is eligible and consents to enroll, they proceed with a baseline visit including the following evaluations: behavioral questionnaire, grip strength test, 15-foot timed walk test, height and weight measurement, blood collection, and anal clinical evaluations (Table 2). The study visit takes ~3 h to complete.

**Table 2:** Research activities performed at each visit, and associated data collected for each Stage 1 and Stage 2 visit, during the AHHA Study.

**Research activities at each visit and data derived as part of activity**	**Stage 1 (Baseline)**	**Stage 2 (6M−36M)**	**Time required (Minutes)**
**Informed consent and overview of study**	X		45
**Baseline questionnaire (self-administered, computer-assisted)**	X		45
Demographics			
Lifetime sexual history			
Lifetime medical history (including HIV related med history)			
Lifetime lifestyle history (smoking, alcohol, recreational drugs,			
exercise)			
Recent sexual history (past 6 months)			
Recent lifestyle history (past 6 months)			
Recent medical history (past 6 months)			
The Fried Frailty Phenotype (FFP)*			
**Follow-up questionnaire (self-administered, computer-assisted)**		X	30
Recent sexual history (past 6 months)			
Recent lifestyle history (past 6 months)			
Recent medical history (past 6 months)			
The Fried Frailty Phenotype (FFP)*			
**Grip-strength test**	X	X	15
The Fried Frailty Phenotype (FFP)*			
**15-foot timed walk test**	X	X	15
The Fried Frailty Phenotype (FFP)*			
**Blood collection**	X	X	15
Markers of inflammation (IL-6, CRP, D-dimer)			
Veterans Aging Cohort Study (VACS) Index (PLWH)**			
CD4+, HIV viral load (PLWH)			
**Anal clinical exam**	X	X	45
Type specific anal HPV DNA			
Histologically confirmed HSIL			
Type specific anal HPV DNA			
**Total baseline time requirement**			**180**
**Total 6M−36M time requirement**			**120**

6M−36M: 6 month to 36-month visits–occurring every 6 months, PLWH, participants living with HIV; IL-6, Interleukin-6; CRP, C-reactive protein.

^*^The FFP is comprised of 5 domains, 3 are assessed by questionnaire, 1 by grip-strength test, and 1 by 15-foot timed walk test.

^**^The VACS Index is comprised of CD4+ level, HIV-1 VL, hemoglobin, Aspartate Aminotransferase (AST) Alanine Aminotransferase (ALT), platelet count, creatinine level, HCV serostatus.

For participants living with HIV (PLWH), we obtain a confirmation of their HIV status from their primary care physician or ask participants to show us a prescription or medication bottle with their name and ART. HIV-negative participants receive a rapid HIV test to confirm their status after appropriate pre-test counseling. Post-test counseling is subsequently performed.

### Self-administered computerized behavioral questionnaire

**Figure 2:**
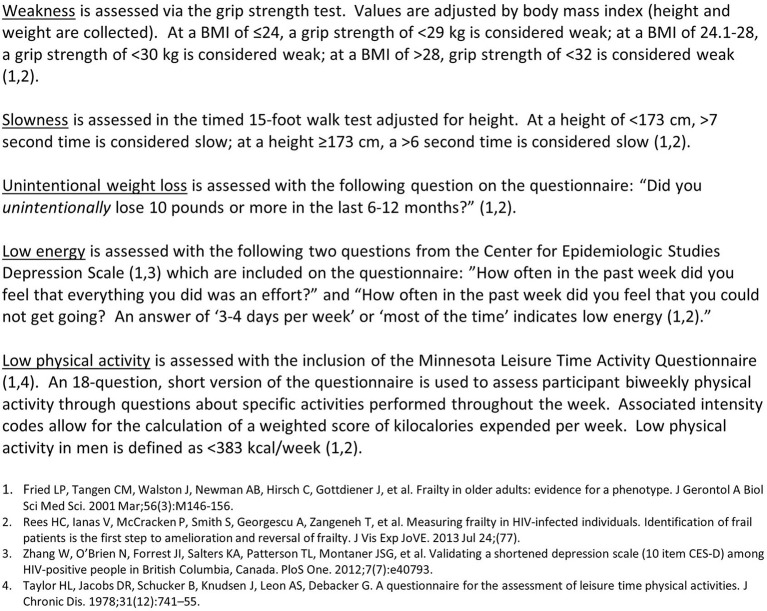
Fried frailty phenotype.

All participants complete a self-administered computerized behavioral questionnaire using REDCap software (33, 34). The questionnaire includes demographic questions on date of birth, race/ethnicity, education, and income, as well as lifestyle questions, including current and history of use of tobacco, alcohol, and recreational drugs. It also includes a brief medical history including: history of anogenital sexually transmitted infection, history of anorectal inflammation, and history of comorbidities such as diabetes or chronic heart disease. A history of sexual behavior is also obtained with questions regarding lifetime and current behaviors, including sex with men and women, sexual practices (including receptive anal intercourse), number of sexual partners, and frequency of sex. Additional questions evaluate the VACS Index and the FFP frailty measure (Figure 2). PLWH are also queried about nadir CD4+ levels, date of HIV diagnosis, history of AIDS and non-AIDS-defining illnesses, history of and current use of ART, and cumulative duration of ART use.

### Grip strength test

Grip strength is a commonly used measure of upper body skeletal muscle function and has been widely used as a general indicator of frailty and is a component of the FFP (25) (Table 1). Grip strength in participants' dominant hand is measured using an adjustable hydraulic grip strength dynamometer (Jamar Hydraulic Hand Dynamometer), which registers maximum kilograms of force during a trial. When the dominant side is affected–e.g., pain in the wrist or hand or if the participant has undergone surgery of the upper extremity in the past 3 months–the grip strength test is performed on the non-dominant hand. If both hands are affected, this examination is skipped. Grip strength is averaged over three trials per visit.

### 15-foot timed walk test

Slowness is a component of the FFP and is assessed in participants with a timed 15-foot timed walk test, adjusted for height (25). This test is not performed if the participant is in a wheelchair or cannot walk 15-feet without assistance. Use of a cane and/or walker is permitted.

A 15-foot area has been measured off and marked in the front hall of the ANCRE Center; additional space is marked at both the beginning and the end of the course, bringing the total distance to 30-feet. Participants are asked to stand with their toes on the first marking. They are asked to walk to the other end of the course at their usual speed, as if they were “walking down the street to go to the store.” Participants begin walking at the first mark, but timing starts and stops at the 15-foot markings. The 15-foot timed walk test is repeated 3 times per visit, starting from the same end each time, and the average of the three walks is used.

### Height and weight measurement

Height in meters (m) and weight in kilograms (kg) are measured by one of the ANCRE medical assistants on a standard scale manufactured by Tanita Corporation, Japan, with shoes removed. BMI is calculated as kg/m^2^.

### Blood collection

All participants have blood collected by a trained phlebotomist to test for immune markers of inflammation (Table 3). We collect 4 milliliters (mL) for interleukin-6 (IL-6), 5 mL for C-reactive protein (CRP), and 1.8 mL for D-dimer from all participants. We also collect 4 mL to test for HPV antibodies which are processed, and the serum is frozen for testing after follow-up is completed for participants diagnosed with incident HPV infection.

**Table 3:** Blood collection volume by test for all participants and those done only for PLWH.

	**Assay**	**Tube volume**	**Visit**	**Total volume baseline**	**Total volume 6M−36M**
HIV-negative participants	HPV antibody	4 mL	Baseline	14.8 mL	10.8 mL
	IL-6	4 mL	Baseline 6M−36M		
	CRP	5 mL	Baseline 6M−36M		
	D-Dimer	1.8 mL	Baseline 6M−36M		
PLWH	HPV antibody	4 mL	Baseline	34.3 mL	30.3 mL
	IL-6	4 mL	Baseline 6M−36M		
	CRP	5 mL	Baseline 6M−36M		
	D-Dimer	1.8 mL	Baseline 6M−36M		
	Complete blood count Hemoglobin* Platelet*	3 mL	Baseline 6M−36M		
	AST* ALT* Creatinine* Hepatitis C Antibody*	5 mL	Baseline 6M−36M		
	CD4+ T-cell level	3 mL	Baseline 6M−36M		
	HIV Viral Load*	8.5 mL	Baseline 6M−36M		

PLWH, Participants living with HIV; AST, Aspartate Aminotransferase; ALT, Alanine Aminotransferase.

^*^Used in compilation of the VACS Index.

PLWH participants have additional blood collection for the compilation of the VACS Index: one additional 3 mL tube for a complete blood count with differential, including hemoglobin and hematocrit, red blood cell count, indices, white blood cell count, and platelet count, 5 mL for creatinine, aspartate aminotransferase (AST), and alanine aminotransferase (ALT), albumin, and hepatitis C virus (HCV) antibodies. HIV CD4+ and HIV viral load (VL) are also collected: 3 mL is collected to test for CD4+ lymphocytes and 8.5 mL to test plasma for HIV VL. Please see Table 3.

For HIV-negative participants, one drop of blood is used to run a rapid HIV antibody test. An HIV antigen/antibody test is performed instead of this test as clinically indicated.

### Clinical evaluations

The AHHA Study physician explains all clinical procedures to the participant before the examination. Participants are advised to have a bowel movement prior to the procedure, if possible, but otherwise not to perform any special cleaning of their anus or colon.

All physicians who perform a digital anorectal examination (DARE) and HRA for the AHHA Study undergo training by ANCRE physicians and meet the standards of the International Anal Neoplasia Society (35).

One tap water-moistened Dacron swab is collected from the anus for anal cytology and HPV DNA testing. The swab is inserted into the anus and rotated ~20–30 times to collect cells and immediately placed in a vial of PreservCyte transport medium and transported at ambient temperature to the lab for testing.

DARE is performed to feel for masses suspicious of cancer.

The clinician then performs an HRA to visualize lesions as described previously (36, 37). Briefly, during an HRA, the anal canal and perianal region are visualized using a colposcope containing a light and magnifier. Five percentage acetic acid and Lugol's solution are applied *via* swab to assist in the assessment for anal lesions (37). Anal biopsies are obtained if lesions are visualized. Typically, a biopsy is obtained from each area with a distinct appearance to find the highest grade lesion present.

Anal cytology and biopsy samples are subsequently sent for pathological analysis performed by the UCSF Pathology department.

### Post visit phone call

Approximately 6 weeks after each visit, the research assistant will call the participant to communicate the results of their anal cytology and anal biopsy. If the participant has anal HSIL, they are referred for appropriate care and thanked for participating in the AHHA Study.

Results of clinical tests are reviewed by the AHHA Study physician. If all clinical results are normal, the nurse communicates that the results are normal. If any of the clinical test(s) have a level not in the normal range, the nurse will communicate that the result was outside of the normal range and that the physician is referring them to their primary care physician for follow-up care. All participants also receive referrals to other care or services when recommended by the AHHA Study physician.

Standard-of-care clinical tests include:

- C-reactive protein (CRP)
- For PLWH:
  ° Creatinine
  ° Estimated Glomerular Filtration Rate (eGFR)
  ° Albumin
  ° Aspartate Aminotransferase (AST)
  ° Alanine Aminotransferase (ALT)
  ° Hepatitis C virus antibody
  ° Complete blood count
  ° CD4+ level
  ° HIV-1 viral load

After the baseline visit, if participants are negative for anal HPV-16 and HSIL, they are included in Stage 2 and scheduled for their next study visit 6 months from the baseline study visit.

## Stage 2—Prospective cohort study to address objectives 2.1–2.3

Participants enrolled in the prospective cohort study (Stage 2) are seen in the clinic every 6 months for 3 years for a total of 6 additional visits (Table 2).

### Follow-up questionnaire

The follow-up questionnaire is based on the baseline questionnaire and collects information on behaviors or medical status that may have changed since the preceding visit 6 months ago. The follow-up questionnaire is the same for each of the follow-up visits.

### Research evaluations

All Stage 2 participants undergo the same research evaluations described for the baseline visit (Stage 1), including the grip strength test, the 15-foot timed walk test, blood collection, and clinical evaluations (Table 2).

## Sample processing for stage 1 and stage 2 studies

All laboratory samples included in Table 3, anal HPV DNA testing, and pathology samples (anal cytology and biopsy samples), are analyzed at UCSF and/or mailed out for analysis to Quest Diagnostics.

### HPV DNA testing of anal swabs

Anal swabs collected in PreservCyt solutions (Hologic, Inc., Marlborough, MA) as part of the clinical examination are tested for HPV DNA to determine the prevalence of anal HPV infection.

HPV testing is performed at the Palefsky Lab at UCSF on de-identified samples. For HPV-positive samples, HPV is genotyped by the Atila AmpFire HPV High Risk Genotyping kit (Cat# GHPVF-100, Atila BioSystems, Mountain View, CA).

Ampfire is an isothermal nucleic acid amplification assay with real time fluorescence detection for qualitative genotyping of 15 high-risk types of HPV (16/18/31/33/35/39/45/51/52/53/56/58/59/66/68). HPV specific primers and fluorescent probes are used to amplify regions of viral genomic DNA, including E6/E7 regions, under isothermal conditions. The assay incorporates an internal control (IC) to verify the adequacy of DNA extraction and amplification stages.

### Amplification and detection of HPV types

To extract DNA, 1 mL of each anal suspension is spun, and the supernatant is removed. Then, each tube receives 100 microliters (μl) of 1X lysis buffer, followed by incubation at 95°C for 20 min. Four pre-labeled tubes receive 12 μl of master reaction mix, and 1 μl of corresponding primer mix per sample, 23 μl of each of the four master mixes, and 2 μl of extracted DNA are added to its corresponding well. For real-time PCR, the isothermal reaction condition is set at 60°C while taking fluorescence reading at FAM/HEX/CY5/ROX channels per minute for 60 min.

### Interpretation of HPV types analyses

For each sample, genotyping results are coded and identified based on exponential amplification curves in the CY5, ROX, FAM, and HEX channels in the above layout of 4 reaction tubes. The lack of an exponential amplification curve of IC in the HEX channel from primer-mix 3 is interpreted as invalid. The negative and positive controls provided in the kit are included in each experiment for quality assurance.

### HPV-16 DNA testing of anal biopsies

In addition to testing anal swab samples, HSIL biopsies are tested for HPV-16 DNA. DNA is extracted from the formalin-fixed paraffin embedded samples, and PCR is performed using standard procedures (38). Participants are only classified as having HSIL for analysis if at least one of their biopsies shows HPV-16 DNA.

### HPV-16 serology

All participants at baseline have blood collected for HPV-16 serology testing. These samples will be stored in a −80°C freezer until completion of Stage 2 follow-up. These samples will then be tested for HPV-16 neutralizing antibodies using the pseudovirion-based neutralization assay, as published previously (39).

### Pathological examination of anal biopsy and cytology results

For this study, biopsy results are used to diagnose participants with HSIL and determine the prevalence or incidence of anal HSIL. Anal histology samples are evaluated by a UCSF pathologist without knowledge of the HIV status, HPV DNA results, or results from other diagnostic tests or questionnaire data. Anal histology results are classified as normal, atypia, LSIL, and HSIL using Lower Anogenital Squamous Terminology (LAST) (40) criteria.

Anal cytology, primarily used for clinical care, serves in the AHHA Study as a quality control measure. Given the high positive predictive value of HSIL-positive cytology for HSIL-positive histology, discordant findings suggest that the clinician missed the lesion and prompts us to ask the participant to return for a repeat HRA (36, 41). Since we require a biopsy that shows HSIL that contains HPV-16 DNA for our incident disease outcome, cytology will not be used in this study to define HSIL status. Anal cytology results will be classified as normal, atypical squamous cells of undetermined significance (ASC-US), atypical squamous cells–cannot rule out high-grade disease (ASC-H), LSIL, HSIL, or cancer using the Bethesda (42) criteria.

## Statistical considerations

### Definitions of key variables

A. A prevalent HPV-16 infection will be defined as HPV-16 detected in the anal swab sample. An incident HPV-16 infection will be defined as HPV-16 detected in anal swab samples in individuals who were HPV-16 negative at baseline. Prevalent and incident HPV infections will be defined similarly for each HPV type examined.
B. Incident HPV-16-associated HSIL will be defined as incident HSIL with HPV-16 DNA detected in the biopsy sample.
C. HIV status is defined by self-report. HIV-positive status is confirmed by medical records or ART medication prescription. HIV-negative status is confirmed by rapid test.
D. Fried Frailty Phenotype (FFP), as defined by Fried (25) and Rees (43) consists of the presence of three or more of five criteria: [weakness (grip strength test), slowness (15-foot timed walk test), unintentional weight loss in the last 12-months (self-report, questionnaire), low energy (self-report, questionnaire), low physical activity in the past 12-month (physical activity, questionnaire)] (Figure 2).
E. Veterans Aging Cohort Study (VACS) Index in PLWH participants. The VACS Index is a weighted combination of age, two markers of HIV disease stage [CD4+ level, HIV-1 VL], five routinely collected clinical tests [hemoglobin, AST, ALT, platelet count, creatinine level, HCV serostatus] (22, 23). At the conclusion of the study, participants will be assigned points on the VACS Index for increasing age values, HIV VL, hemoglobin, Fibrosis-4 index, decreasing values of CD4+ level and eGFR, and positive HCV serostatus (22). A higher value on the VACS Index (range, 0–163) indicates poorer health status. The AHHA Study does not collect blood for CD4 or VL from HIV-negative participants; thus, this measure of biological aging cannot be compared between HIV+ and HIV- cohorts.

### Objective 1 analyses

#### Type-specific HPV infection

Using generalized estimating equation (GEE) models with robust standard errors, we will estimate the mean (95% CI) presence of type specific anal HPV infection. Associations of risk factors for HPV-16 infection will be estimated *via* odds ratios (OR; 95% CI) in a multivariable GEE model: HIV status, chronological age (50–59, 60–69, 70+ years), the interaction between age groups and HIV status, and biomarkers of inflammation (plasma levels) and aging (FFP and VACS Index). OR (95% CI) will be illustrated *via* a forest plot. Similarly, we will study the presence of any HPV infection. Exploratory analyses may evaluate associations with personal characteristics–including race, BMI, education, income, sexual history, tobacco use, alcohol use, and use of recreational drugs.

In a subgroup analysis of PLWH, the following HIV-related characteristics will replace HIV status in the model of anal HPV-16 or any HPV infection: nadir CD4+ levels, history and current use of ART, duration of ART use, use of any protease inhibitor or NNRTI, duration of HIV diagnosis at AHHA Study enrollment, history of AIDS-defining illnesses and non-AIDS-defining illnesses.

#### Anal HSIL

We will follow the approach above to estimate the mean (95% CI) presence of anal HSIL by HIV status and to evaluate associations of age, biomarkers for inflammation, and biomarkers for age, as risk factors for anal HSIL. An extended model will estimate associations of risk factors for HSIL, including the presence of HPV infection. We will also determine if age and HIV status are effect modifiers of the relationship between each marker and HPV infection. In exploratory models, risk factors to be screened for univariate associations include race, education, income, sexual history, tobacco use, alcohol use, and use of recreational drugs.

In a subgroup analysis of PLWH, HIV-related characteristics will replace HIV status terms in the model of anal HPV-16 as described for Objective 1.1.

### Objective 2 analyses

#### Anal HPV-16 incidence

The Kaplan-Meier method will be used to estimate the 3-year incidence density (95% CI) of HPV-16 by HIV status, adjusted for the age group. Time-dependent Cox regression methods will be used to analyze incident HPV-16 infection from baseline as a function of HIV status, and biomarkers of aging measured over time at follow-up study visits. Time will be specified in calendar months. Results will be summarized as relative hazards with associated 95% CIs.

#### Anal HSIL incidence

This study outcome will be analyzed as described for anal HPV-16 incidence.

### Power and sample size considerations

#### Stage 1

Preliminary estimates of baseline prevalence of HPV and HSIL suggest that HSIL will be found in in 20 and 50% of the HIV-negative and PLWH cohorts, respectively. Sample size calculations assuming *n* = 150 per cohort identify OR = 2.5 for HSIL and OR = 2.0 for HSIL as detectable minimum thresholds for 2-sided 0.05-level tests with 80% power. Initial models will be extended to include biomarkers (IL-6, CRP, D-dimer, and FFP). Thus, the Stage 1 sample has high power to achieve the stated objectives.

#### Stage 2

Preliminary estimates of baseline prevalence of HPV and HSIL suggest that 60% of the Stage 1 HIV- cohort will be eligible for Stage 2 longitudinal follow-up compared with 30% of Stage 1 HIV+ cohort. We anticipate that ~85% of eligible participants will consent to longitudinal follow-up (e.g., 90 HIV- and 45 PLWH; Figure 1), as we have found in other ANCRE clinic studies.

**Figure 3:**
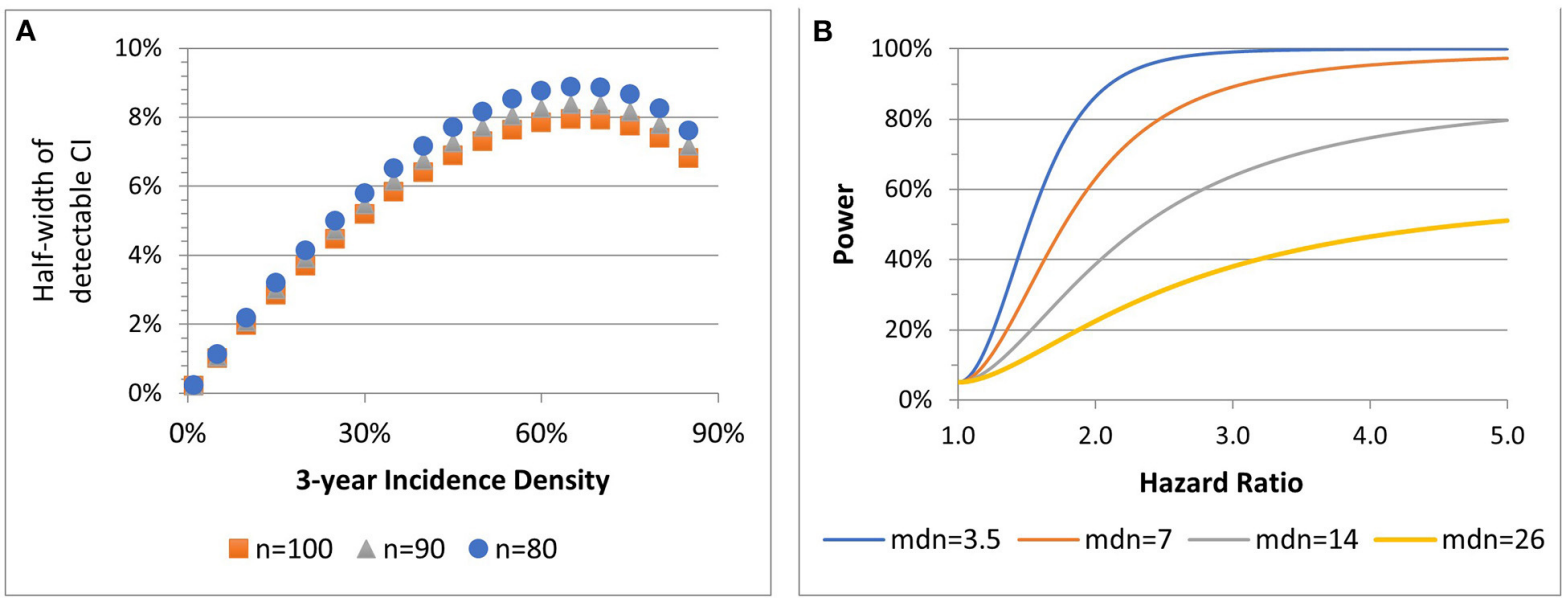
Stage 2 precision associated with 90% power to estimate incidence density for three sample sizes (n) **(A)**. Power of 2-sided 0.05-level test to detect hazard ratios, by median (mdn) time to outcome among HIV- participants at total *n* = *135* **(B)**.

Figure 3A shows the precision with which 3-year incidence densities between 1 and 90% can be estimated for three total sample sizes per cohort, assuming HIV-negative:PLWH cohort sizes are 2:1. For example, if the 3-year incidence density of HSIL among PLWH in Stage 2 is 10%, the confidence interval width would be ±2%. The lowest precision is near 65% incidence (which is unlikely). Cox regression models will be used to assess associations of outcomes with age and biomarkers (IL-6, CRP, D-dimer, and FFP).

Figure 3B presents four power scenarios for two-sided 0.05-level log rank tests. Power increases with a shorter median time to event among HIV-negative participants and with an increasing hazard ratio contrasting HIV status (positive:negative) or any other risk factor. Power is highest when comparison group sizes are balanced. If the median time to event is 3.5 years among HIV-uninfected, the sample has 86 and 99% power to detect HR = 2 and HR = 3. For 2:1 low- to high-risk, if the median time to event is 3.5, the sample has 82 and 98% power to detect HR = 2 and HR = 3.

## Discussion

Anal cancer is now one of the most common cancers among MSMLWH (5). Unlike most other cancers in PLWH, anal cancer is potentially preventable through vaccination (44) or *via* treatment of HSIL (12). Therefore, understanding the relationship between aging, HPV, and HIV in the pathogenesis of anal cancer is of great importance. As the population of PLWH aged 50+ increases, the public health community is confronted with the potential for a vast increase in the number of individuals with anal cancer. It is crucial that we first characterize the extent of the problem in individuals over 49 years and identify potential biomarkers that could be used to identify high-risk populations to target with screening and/or treatment interventions.

Our study is the first that we are aware of prospectively investigating anal HPV related disease in relation to aging and HIV disease among this older population. We will be studying PLWH and HIV-participants to evaluate whether differences are due to HIV status or aging. Our study will also be the first to investigate both the associations between biomarkers of inflammation (IL-6, CRP, and D-dimer) and HPV infection and disease. Additionally, because it is not known if the causal pathway from aging to cancer occurs only through immune system dysfunction, we will also include two composite biomarkers of biological aging that include other systems, the VACS Index and the FFP. We expect that study results for our primary objectives will be directly relevant to clinicians and public health professionals when deciding on screening and treatment options for their older patients and for their MSM and trans woman patients.

We anticipate a number of potential challenges. Our population is hard-to-reach and not easily identifiable in available standardized records or methods commonly used to recruit younger MSM. Older MSM or trans women may also spend more time at home than at public venues typically used to target more youthful men. Similarly, it may be difficult for older individuals to attend a 3-year follow-up study, which may impact our retention rate. While we are initially accounting for up to 15% loss-to-follow-up in our sample size, we may have greater loss-to-follow-up than anticipated. Our recruitment and retention specialist focuses on finding and maintaining relationships with each participant to minimize both of these limitations.

As we ask participants to recall behaviors over their lifetime–and our population is over 50–there may be some recall errors in variables determined by self-report. We have attempted to minimize this by providing visual representations of timelines and computer prompts to help participants answer questions that include time (e.g., how many male partners did you have when you were between the ages of 20–29?) However, we expect that some imprecision within the data will be inevitable. If there is any differential recall with chronological or biological age and age, in turn, is associated with HSIL status, we may have misclassification of behaviors among the group of individuals with HSIL. This misclassification may be in either direction as participants may both over- or under- estimate behaviors with poor recall. We have sought to minimize this by including categorical responses instead of a numerical response hoping that a range will be easier to recall than a specific number. For example, we ask “How may male partners did you have when you were between the ages of 20–29” and the response options are: 0, 1, 2–49, 50–99, 100–249, etc. As age is associated with most deleterious health outcomes and may impact recall of many self-reported behaviors, this type of bias impacts most studies of aging, self-reported behaviors, and health outcomes.

The findings of this study will provide new insight into the role of aging and HIV in the pathogenesis of HPV-related anal cancer. Our data will also have important clinical implications. This data will allow us to understand the need for screening and clinical monitoring among men in different age cohorts.

## Ethics statement

This protocol was approved by the University of California, San Francisco Institutional Review Board (IRB: 16-18966). Participants provided written informed consent.

## Author contributions

AH and JP designed the study. AH prepared the manuscript. AH and JH prepared the preliminary statistical analysis section. CW and RG edited and updated the protocol. JK organized and consulted on clinical evaluation portions of the protocol. CW, RG, JC, and AA helped develop non-clinical research evaluation and prepared corresponding sections of the manuscript. MD and SF developed laboratory protocols. All authors reviewed and contributed to the manuscript.

## Funding

This work was supported by the National Cancer Institute of the National Institutes of Health, Grant Number: 1R01CA206477-01.

## Conflict of interest

The authors declare that the research was conducted in the absence of any commercial or financial relationships that could be construed as a potential conflict of interest.

## Publisher's note

All claims expressed in this article are solely those of the authors and do not necessarily represent those of their affiliated organizations, or those of the publisher, the editors and the reviewers. Any product that may be evaluated in this article, or claim that may be made by its manufacturer, is not guaranteed or endorsed by the publisher.
